# The rhythm of sensory input shapes audio‐visual temporal processing

**DOI:** 10.1111/bjop.70029

**Published:** 2025-09-16

**Authors:** Denisa Adina Zamfira, Giuseppe Di Dona, Gianluca Marsicano, Martina Battista, Luca Battaglini, Luca Ronconi

**Affiliations:** ^1^ School of Psychology Vita‐Salute San Raffaele University Milan Italy; ^2^ Division of Neuroscience IRCCS San Raffaele Scientific Institute Milan Italy; ^3^ Department of Psychology and Cognitive Science University of Trento Rovereto Italy; ^4^ Psychology Program, Division of Science New York University Abu Dhabi Abu Dhabi United Arab Emirates; ^5^ Centre for Studies and Research in Cognitive Neuroscience University of Bologna Cesena Italy; ^6^ Dipartimento di Psicologia Generale University of Padova Padova Italy; ^7^ Neuro.Vis.U.S. Laboratory University of Padova Padua Italy; ^8^ Centro di Ateneo Dei Servizi Clinici Universitari Psicologici (SCUP), University of Padova Padova Italy

**Keywords:** audio‐visual processing, frequency, multisensory integration, rhythmic sensory stimulation, speech, temporal binding window (TBW)

## Abstract

The temporal relationship between incoming signals is crucial in determining whether multisensory information is integrated into unitary percepts. Temporal binding windows (TBWs) define the time range within which multisensory inputs are highly likely to be perceptually integrated, even if asynchronous. TBWs widen with stimulus complexity and neurodevelopmental conditions (e.g., autism and schizophrenia), yet the key factors underlying their malleability remain unclear. The (quasi)rhythmic properties of sensory inputs, frequently embedded in natural stimuli (e.g., speech), are among the possible exogenous modulators. Indeed, stimulus spectral features can influence the alignment of neural excitability across sensory regions, synchronizing brain rhythms with external rhythmic patterns through phase‐reset mechanisms and neural entrainment. In a series of psychophysical studies, we presented simultaneity judgement tasks with pulsing audio‐visual (AV) streams amplitude‐modulated according to different regular frequencies or following purely rhythmic vs. quasi‐rhythmic (speech‐like) envelopes. Results show that TBWs decrease as the stimulus frequency increases and that speech‐like streams are integrated across larger TBWs. These findings highlight the importance of stimulus spectral structure in shaping multisensory perception. Furthermore, they show that quasi‐rhythmic spectrotemporal features of speech‐like streams induce more tolerant cross‐modal temporal processing even when the leading stimulation frequency is controlled for, putatively reflecting an adaptation to the variable rhythmic structure of natural speech. Our results align with neurophysiological accounts of neural entrainment and motivate future research in clinical populations with multisensory processing deficits.

## BACKGROUND

We live in a world rich in multisensory stimuli, and our brain needs to integrate and segregate cross‐modal information in space and time to construct a coherent and smooth representation of the environment (Grassi & Casco, 2010; Spence, 2007; Stein & Stanford, 2008; Wallace & Stevenson, 2014; Zhou et al., 2020). A relevant area in the study of multisensory perception aims at clarifying how the brain groups these inputs coming from different sensory modalities and how unified percepts are generated. According to the *assumption of unity*, the more amodal properties are shared by multisensory information, the more this information would be attributed by the brain to the same physical source in the environment (Vroomen & Keetels, 2010; Welch et al., 1986; Welch & Warren, 1980).

Among amodal factors, the perception of time and, in particular, synchrony between the sensory inputs is not straightforward, as there is no dedicated sense organ for perceiving time on an absolute scale. Instead, all sensory modalities are possible entry points at the interface of physical time with perceptual time (Wittmann, 2009). For instance, light travels through air much more rapidly than sound. Such discrepancies in propagation times are partially compensated by the biophysical properties of our sensory receptors, since the mechanical transduction of sound waves in the ear takes considerably less time than is required for the chemical transduction of light in the retina (Fain, 2003). Accordingly, the temporal coincidence between multisensory inputs plays a crucial role in leading to the perception of multisensory events as unified percepts (Recanzone 2009; Fain, 2003; Murray et al., 2016; Pasqualotto et al., 2016). For instance, when speech perception occurs in a noisy environment, seeing a speaker's face and mouth can greatly help us to understand what the speaker is saying (Bishop & Miller, 2009; Munhall & Vatikiotis‐Bateson, 2004; Van Wassenhove et al., 2007). This happens because the speaker's facial movements carry information not only on ‘what’ the speaker is saying but also on ‘when’ the speaker is saying it (Grant & Seitz, 2000; Vatakis et al., 2008; Vatakis & Spence, 2008; Venezia et al., 2016; Vroomen & Stekelenburg, 2011). More broadly, multisensory perception is facilitated when sensory streams are coherent across both space and time (Murray et al., 2016). Such coherence enables the brain to generate accurate predictions about the identity and timing of incoming information, thereby enhancing perceptual accuracy and efficiency.

However, a plethora of studies on multisensory integration has found that our brain shows a certain temporal tolerance (Andersen et al., 2004; Vatakis & Spence, 2008), which allows us to bind multisensory information even in case of considerable time lags between stimuli (i.e., stimulus onset asynchronies—SOAs), as long as they are presented within the so‐called ‘horizon of simultaneity’, better known as the temporal binding window (TBW) (Colonius & Diederich, 2004; Noel et al., 2016; Pasqualotto et al., 2016; Stevenson et al., 2017; Zampini et al., 2005). Thus, multisensory temporal integration is still possible over a wide time window of hundreds of milliseconds, and the width of TBWs is generally used as an index of audio‐visual (AV) temporal acuity (i.e., the ability to correctly integrate sensory stimuli coming from the same external source and segregate stimuli from different sources). Narrower TBWs typically represent better temporal acuity, while wider TBWs are indicative of higher temporal tolerance when binding different sensory stimuli, with the risk of merging stimuli that are not part of the same environmental event (Colonius & Diederich, 2004; Hairston et al., 2005; Marsicano et al., 2022, 2023; Noel et al., 2016; Pasqualotto et al., 2016; Roach et al., 2011; Zampini et al., 2005).

Such audio‐visual temporal tolerance compensates for the latency differences in propagation times, characterizing the auditory and visual sensory domains (King & Palmer, 1985; Pöppel et al., 1990). Having a relatively wide TBW is beneficial for the integration of multisensory stimuli, since it makes us insensitive to small differences in the arrival time of signals to different sensory modalities (Eg et al., 2015; Noel et al., 2016; Remez et al., 2008; Spence, 2007; Spence & Squire, 2003; Stevenson et al., 2014). Increasing evidence has shown that the width of TBWs in multisensory processing varies significantly among healthy individuals (Ferri et al., 2017; Stevenson et al., 2012) and even more across the continuum from healthy to clinical conditions (Ferri et al., 2018; Marsicano et al., 2022). Moreover, atypical TBWs representing dysfunctional integrative mechanisms have been reported for clinical conditions such as autism, schizophrenia and dyslexia, and have been linked to perceptual and communicative impairments (Ferri et al., 2017, 2018; Hairston et al., 2005; Marsicano et al., 2022; Stevenson et al., 2012; Wallace & Stevenson, 2014; Zhou et al., 2020).

It is worth noting that the width of the audio‐visual TBW is largely modulated, among different factors (i.e., temporal and spatial coincidence, subjective salience and predictability), also by the intrinsic characteristics of the multisensory inputs to be bound (Bean et al., 2021; Munhall & Vatikiotis‐Bateson, 2004; Pasqualotto et al., 2016; Zierul et al., 2019). Specifically, given that the type of multisensory stimuli we encounter in daily life is quite heterogeneous in terms of complexity, their TBW also shows a modulation in width, which reflects different temporal tolerance to stimuli onset asynchronies. Previous studies within the audio‐visual domain have found that simple flash and beep pairs are integrated in narrower windows, which progressively get wider with more complex ecological stimuli, reaching the widest size with speech, one of the most complex naturalistic AV stimuli (Conrey & Pisoni, 2006; Miller & D'esposito, 2005; Stevenson & Wallace, 2013; van Atteveldt et al., 2007; Van Wassenhove et al., 2007; Vatakis & Spence, 2008). Although a certain degree of plasticity in TBWs emerged across studies, the key parameters that determine this malleability have been poorly explored. With this regard, a relevant aspect when considering multisensory interaction in a naturalistic environment is that multisensory events (e.g., speech) are typically continuous and dynamic, with intrinsic rhythmic (or quasi‐rhythmic) properties (Kohler, 2009; Meyer et al., 2020). Thus, when investigating audio‐visual integration with more ecologically relevant stimuli, the contribution of these intrinsic features and, consequently, different degrees of complexity to the process of multisensory temporal binding needs to be considered. Recent findings have strengthened this idea, showing that even for complex auditory streams that do not carry any linguistic content, the coupling with a concurrent congruent visual stream improved the analysis of the audio stream itself (Atilgan et al., 2018; Maddox et al., 2015). Specifically, participants show significantly better behavioural performance when they are presented with a video that is synchronized with the target auditory stream, while the performance drops when the video is synchronized with an irrelevant background sound (Maddox et al., 2015). Natural audio‐visual streams, like speech, have a quasi‐rhythmic structure that impacts on cross‐modal integration and could have cascade effects not only on behavioural performance but also on higher order cognition (Bauer et al., 2020; Doelling et al., 2014; Dumas et al., 2010; Lakatos et al., 2008, 2019; Ronconi et al., 2016; Wass et al., 2020; Yun et al., 2012). With this regard, psychophysics evidence shows that multisensory temporal acuity is increased for unpredictable/arrhythmic sequences relative to predictable/rhythmic ones (Denison et al., 2013). Moreover, the slow amplitude modulations of speech carry quasi‐regular rhythmic information, fundamental for speech comprehension (Meyer et al., 2020; Poeppel & Assaneo, 2020); removing or degrading it from the spectrum decreases speech intelligibility (Peelle & Davis, 2012). Thus, the quasi‐rhythmic properties of speech might be considered a contributing parameter that adds complexity to the speech signal, accounting for wider TBWs.

Clarifying what is the impact of specific frequencies of multisensory information on perceived synchrony and on temporal integration is a relevant factor in order to address whether changes in our temporal acuity might derive from congruent cross‐modal modulation of these events operated by central supramodal neurocognitive mechanisms. Fujisaki and Nishida (2005, 2007) provided the first evidence that AV integration for rhythmic stimuli is mediated by a slow central process that combines properties of a single event; in particular, they investigated whether human perception of AV synchrony is influenced by the frequency of presentation of the AV stream, and whether and to which extent there is a limit in human synchrony perception. Their findings suggest that the limit of AV synchrony versus asynchrony discrimination is 4–5 Hz, regardless of which visual and auditory attributes are combined. When the frequency of AV stimuli presentation exceeded the 4–5 Hz limit, the synchrony–asynchrony discrimination became nearly impossible for participants, even when the AV interval was sufficiently wide (Benjamins et al., 2008; Fujisaki & Nishida, 2007). This frequency matches well with the frequencies of slow amplitude modulations in natural speech (Poeppel & Assaneo, 2020).

In the present study, we hypothesized that rhythmic and quasi‐rhythmic properties of AV information could partly account for changes in our ability to correctly integrate or segregate information in the environment, and thus for the malleability of TBWs. To test our hypothesis, we conducted a web‐based data collection on a large sample of participants. We used a series of simultaneity judgement (SJ) tasks in order to measure individual TBWs during the presentation of ‘pulsing’ AV streams characterized by a congruent modulation in amplitude (for sounds) and size (for visual stimuli). In the first two experiments, we investigated whether the frequency of regular rhythmic AV streams (1 Hz vs. 2 Hz vs. 3 Hz) had an impact on multisensory integration. The aim was to understand whether the intrinsic spectral properties embedded in AV streams could be among the critical factors responsible for the widening of TBWs. Then, we conducted a third experiment to compare audio‐visual integration for regular (rhythmic) and irregular (i.e., quasi‐rhythmic/speech‐like) AV streams (3 Hz). The latter were created by modulating the amplitude of the AV components following a complex function obtained from the speech envelopes of real speech signals. By holding stimulus frequency constant, we specifically isolate the contribution of envelope regularity to AV integration/segregation. We hypothesized that the quasi‐rhythmic structure of speech‐like streams would result in wider TBWs, reflecting an adaptation to the variability and complexity of natural communication signals.

## EXPERIMENT 1

In Experiment 1, we tested whether AV temporal acuity might be modulated when processing rhythmic AV streams presented at different frequencies. We selected three frequencies (i.e., 1, 2 and 3 Hz) that were below the limits of human AV synchrony judgements (i.e., 4–5 Hz) (Benjamins et al., 2008; Fujisaki & Nishida, 2007). Thus, we implemented an online version of the Simultaneity Judgement (SJ) task to extract individual TBWs, which we used as a proxy for AV integration and temporal acuity. Moreover, to explore any differences in AV integration/segregation processes within the two sensory modalities involved (vision and hearing), we compared participants' perceived simultaneity as a function of the leading sense, that is, auditory leading, where the auditory stream was presented first (AL), and visual leading (VL) where the visual stream was presented first. This analysis allowed us to assess any potential asymmetries in the processing of AV rhythmic streams.

### Materials and methods

#### Participants

An initial sample of 100 participants (*F* = 70; mean age = 22.29) was recruited among university students through advertisement and word of mouth. Participants did not receive compensation or course credits. All were volunteers and presented normal or corrected to normal vision and hearing. Exclusion criteria were self‐reported neurological disorders and epilepsy/photosensitivity. Data from 5 subjects were excluded because they did not complete the task. Moreover, datasets of participants that did not reliably fit the Gaussian psychometric function (Adjusted *R* ^2^ < .5) or the logistic function (Adjusted *R* ^2^ < .3) were excluded from further analyses (see Results section for information on the final sample sizes for each analysis) following the same procedures used in previous similar studies (e.g., Bedard & Barnett‐Cowan, 2016; Hillock‐Dunn et al., 2016; Marsicano et al., 2022). Detailed instructions about the procedure to correctly perform the task were given during the recruitment process, and additional information was implemented in the web‐based task. The study was conducted in accordance with the Declaration of Helsinki and approved by the Ethical Committee for Psychological Research of the University of Padova (Protocol number 4026). All participants gave their informed consent.

#### Stimuli and procedure

**FIGURE 1:**
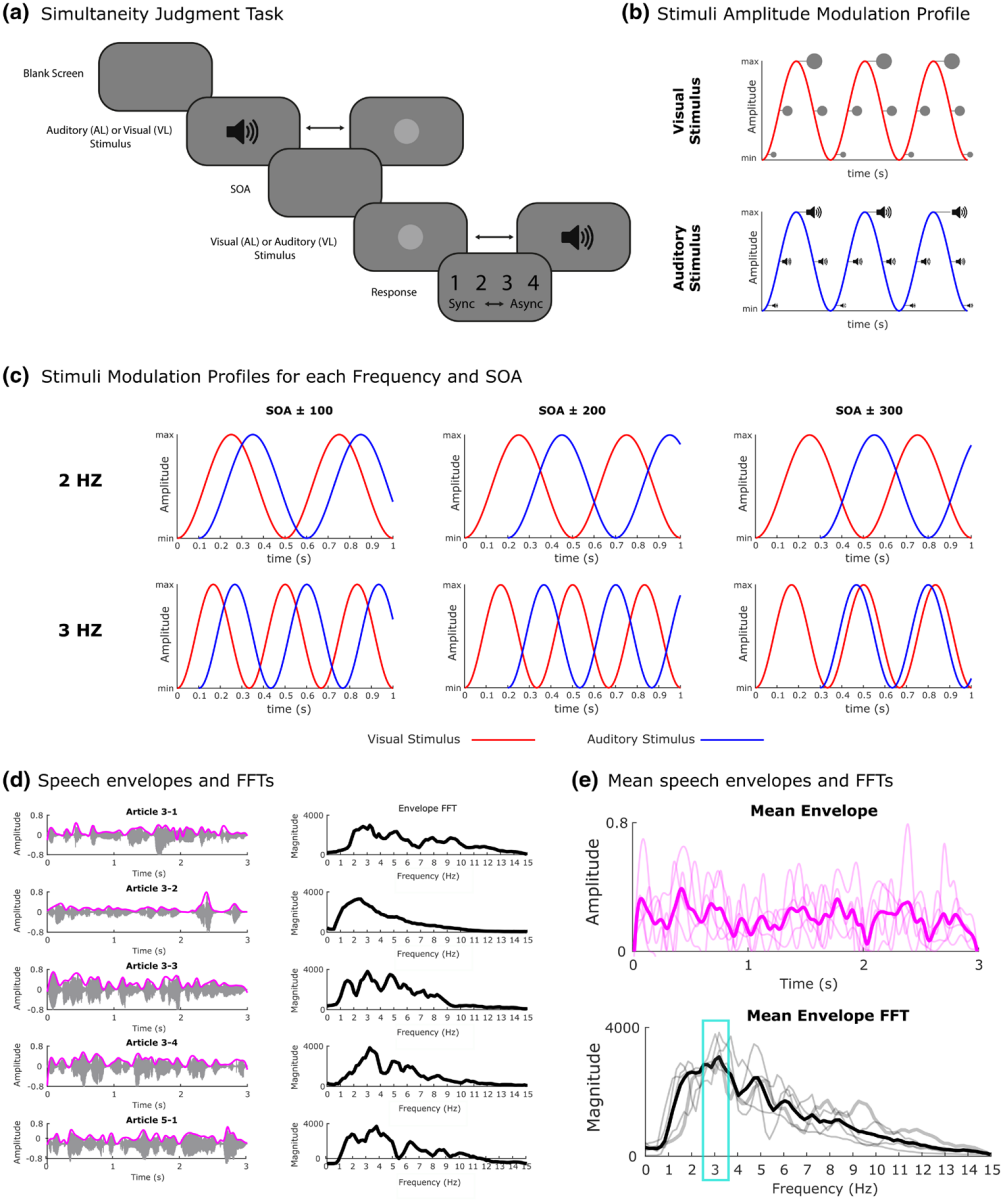
Experimental task and audio‐visual stimuli. Schematic representation of the Simultaneity Judgement (SJ) Task employed in Exp. 1, 2 & 3 (a). Exemplary modulation profile of the circle size employed as visual stimulus (red) and of the amplitude of the modulated tone employed as auditory stimulus (blue) (b). Exemplary modulation profile for the AV streams (red = visual stream, blue = auditory stream) for each combination of frequency and SOA. The examples only show the visual‐leading (VL) condition, but the same rationale applies to the auditory‐leading (AL) condition (c). Amplitude profiles (grey) of the recorded speech tokens whose envelopes (pink line) were used to modulate the auditory and visual streams of the speech‐like condition in Exp 3 (left column) and the FFTs of the envelopes are represented for each token (right column) (d). Mean envelope and FFT computed across all stimuli (e).

Dynamic and continuous auditory and visual streams, consisting respectively of a pure tone (500 Hz) and a grey Gaussian blob with a diameter of 6 deg, were presented on a black background at the centre of the screen. Stimuli were coherently modulated in amplitude and size, following a sinusoidal function. Specifically, the audio‐visual stream had a duration of 3 s, and it was created such that the sound varied in its amplitude continuously. At the same time, the visual stimulus continuously varied in its size (i.e., radius) congruently with the amplitude variation of the sound. Audio‐visual stimuli were presented at 11 stimulus onset asynchronies (SOAs: ±500, ±400, ±300, ±200, ±100, 0 ms), with either the auditory or the visual stimulus first. Negative SOAs represent trials where the auditory stimuli were presented before the visual stimuli (i.e., auditory leading, AL) while positive SOAs represent trials where the visual stimuli were presented before the auditory ones (i.e., visual leading, VL) (see Figure 1 for a schematic representation of the task and stimuli).

Additionally, stimuli could be presented at three different stimulation frequencies: 1, 2 and 3 Hz. Thus, the audio‐visual stream had not only the same frequency characteristics, but was created so that the spatio‐temporal variations of the AV stimuli would take place in the range which is known to characterize speech amplitude envelope and mouth movement, that is, between 2 and 7 Hz (Chandrasekaran et al., 2009; Maddox et al., 2015). Moreover, considering that the audio‐visual stimuli had a duration of 3 s, the number of pulses varied on the basis of the frequency condition. Specifically, the number of pulses was 3, 6 and 9 for the 1, 2 and 3 Hz conditions, respectively (see Figure 1).

Before beginning the task, participants had to fill out a computer‐based questionnaire assessing their demographics (e.g., age, sex and years of education). Then, after a brief familiarization with the SJ task through three example streams (SOA = 0, SOA = −500 for the AL condition and SOA = 500 for the VL condition), they were prompted by on‐screen instructions to start the experiment by pressing the spacebar. Each trial started with a central fixation cross lasting for 500 ms, followed by the presentation of the AV streams. The auditory stream was presented binaurally, and the visual stream was presented at the centre of the screen.

The total number of experimental trials was 165, which were presented in a single block (five repetitions for each combination of SOAs and frequencies). The trials were randomly administered across participants. In order to assess whether the audio‐visual streams pair was perceived as synchronous or not, at the end of each trial, participants were asked to report, without time constraints, the rate of perceived simultaneity through a 5‐point scale, by selecting through the keyboard one of the following five options: 1 = certainly synchronous; 2 = probably synchronous; 3 = not sure; 4 = probably asynchronous; 5 = certainly asynchronous. Thus, subjective audio‐visual synchrony was attributed to responses 1 and 2, and subjective audio‐visual asynchrony to responses 4 and 5. The ‘not sure’ answer option was included to allow participants to express genuine uncertainty without being forced into an arbitrary decision, but it was excluded from the analysis because it was not informative about the perceived synchrony or asynchrony of the audio‐visual streams.

The SJ task was implemented in Psychopy (Peirce, 2007), through Builder GUI, and, after being transformed into PsychoJS, it was administered on Pavlovia (https://pavlovia.org/), an online platform for psychophysics experiments. Participants received a link along with instructions on how to properly undergo the web‐based task. The task could be executed only via PC and, given the lack of supervision typical of controlled laboratory settings, we strongly recommended that participants execute the task in a dimly lit and quiet room. All stimuli of the experimental paradigm were optimized for a 60 Hz monitor refresh rate. We collected information about the type of OS used by the participants (Windows = 73 participants, MacOs = 22 participants). We asked subjects to run the experiment using Mozilla Firefox or Google Chrome as web browsers. During an extensive pilot observation, it has been shown that such browsers are the most reliable in terms of stimulus presentation across different OS. We underlined the importance of following the instructions we provided for an optimal execution of the online task. We emphasized the critical importance of sitting in a dimly lit and quiet room, using earphones at a comfortable volume, and keeping a viewing distance of ~50 cm from the screen. The use of a 60 Hz refresh rate monitor during the execution of the task, which ensured a correct timing of stimulus administration, was confirmed for all 100 participants, based on log file information.

### Data analysis

For each participant, we first computed the proportion of simultaneity rates in function of SOAs. Then, we fitted each individual distribution of responses to a Gaussian function via the Curve Fitting toolbox implemented in MATLAB 2020a (MATLAB, 2020) by using the following formula:

$$y=a*e^{-{\left(\left(x-b\right)/c\right)}^{2}}$$

In this equation, *x* represents the SOAs (ranging from −500 to +500 ms, in steps of 100 ms) between the auditory and visual stimuli, while *y* represents the proportion of perceived simultaneity (ranging from 0 to 1) reported from participants at the SJ task. The *a* parameter represents the peak amplitude of the Gaussian curve on the *y* axis, while the *b* parameter represents the centroid of the peak on the *x* axis, that is, the SOA at which the auditory and visual stimuli are perceived as being maximally simultaneous, also known as the point of subjective simultaneity (PSS). The last parameter, *c*, represents the standard deviation or the width of the curve, that is, the time range (in ms) within which the audio‐visual streams were subjectively perceived as simultaneous despite being physically asynchronous, also referred to as TBW. During the fitting procedure, the upper *a* bound was set at 1 while the *b* and *c* parameters were left free.

In addition, we fitted each individual distribution of responses also to a logistic function, separately for the AL (SOAs from −500 to 0 ms) and VL (SOAs from 0 to +500 ms) conditions. The following equation was used:

$$y=1/\left(1+e^{\left(b*\left(t-x\right)\right)}\right)$$

where *x* represents the SOAs between the auditory and visual stimuli while *y* represents the proportion of perceived simultaneity reported from participants at the SJ task. In this case, the *b* parameter represents the curve's slope (or steepness) and ranges between 0 and 1 for the AL condition and between −1 and 0 for the VL condition. Thus, during the fitting procedure, the lower bound of the *b* parameter was set to 0 for the AL condition while its upper bound was set to 0 for the VL condition. The *t* parameter represents instead the 75% threshold (i.e., the amount of onset asynchrony required to elicit a perceived simultaneity in 75% of cases). No constraints were applied for this last parameter. Since for the VL condition the perceived simultaneity decreases as the SOA increases, resulting in negative slopes, we considered the absolute values of the *b* parameter for subsequent analyses. Similarly, the thresholds for the AL condition are negative, so we considered the absolute values of the *t* parameter.

For the statistical analyses, a repeated measures Analysis of Variance (rm‐ANOVA) was conducted on SJ rates with stimulus frequency (1, 2 and 3 Hz) and SOA (−500, −400, −300, −200, −100, 0, +100, +200, +300, +400 and +500 ms) as within‐subject factors. This first analysis aimed to test whether the perception of synchrony was influenced by the frequency and the SOAs at which the AV stimuli were presented. A second rm‐ANOVA on TBWs (i.e., the *c* parameter from the Gaussian fitting) was conducted using stimulus Frequency (1, 2, 3 Hz) as a within‐subject factor. Finally, two different rm‐ANOVAs on slope and threshold values extracted from the logistic fitting were conducted to test differences in AV integration/segregation processes as a function of the leading sense. In these last cases, we included stimulus frequency (1, 2 and 3 Hz) and leading sense (AL and VL) as within‐subject factors.

Prior to statistical analyses, variables were plotted and tested for normality using the Shapiro–Wilk test. The Greenhouse–Geisser correction was applied in cases in which sphericity was violated (Mauchly test *p* < .05). All the statistical analyses were conducted using R Statistics (R Core Team, 2020) with customized scripts. The ANOVAs were performed using the ‘ez’ package, and post hoc comparisons were conducted using paired‐sample *t*‐tests included in the base ‘stats’ package. To control for multiple comparisons, the Bonferroni procedure was applied. The significance level (alpha) for all analyses was set at .05.

### Results

Among the 95 subjects who completed the task (68 F, mean age = 22.26, SD = 2.1, age range = 20–31), data from seven participants were excluded from the analyses on TBWs and SJ rates because of a poor fitting of the Gaussian function (adjusted *R* ^2^ < .5). We opted to keep this outlier identification criterion also for the ANOVA on SJ rates because we ascertained that participants who had a bad Gaussian fit had performed poorly at the SJ task. Thus, the final sample for these analyses included 88 subjects (61 F, mean age = 22.34, SD = 2.11, age range = 20–31). For the analyses on measures extracted from the logistic fitting (i.e., *b*/slopes and *t*/thresholds), a lower goodness of fit criterion was used to identify potential outliers (adjusted *R* ^2^ < .3 in either the AL or VL condition). The final sample in this case included 73 subjects (50 F, mean age = 22.32, SD = 2.15, age range = 20–31).

**FIGURE 2:**
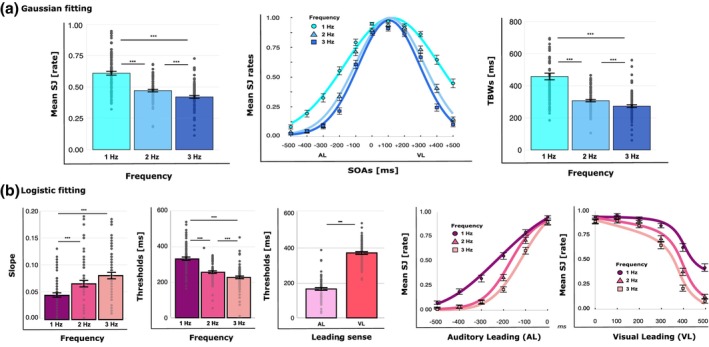
Experiment 1 results. (a) Gaussian fitting results: Mean simultaneity judgements (SJ) and the temporal binding windows (TBWs) with error bars (SEM) and individual data points for each stimulation frequency (1 Hz = cyan, 2 Hz = light blue, 3 Hz = dark blue) and SOA (1 Hz = circles, 2 Hz = triangles, 3 Hz = squares). (b) Logistic fitting results: Mean slope and threshold values with error bars (SEM) and individual data points for each stimulation frequency (1 Hz = violet, 2 Hz = fuchsia, 3 Hz = dark pink), leading sense (AL = light pink, VL = red) and SOA (1 Hz = circles, 2 Hz = triangles, 3 Hz = squares). Asterisks indicate the level of statistical significance (***p < .001, **p < .01).

The rm‐ANOVA on SJ rates showed a significant main effect of frequency (*F*_(1.48,129.46)_ = 109.18, *p* < .001, η^2^ = .56), SOA (*F*_(4.15,361.04)_ = 470.35, *p* < .001, η^2^ = .84) and a significant Frequency × SOA interaction (*F*_(12.21,1062.14)_ = 12.22, *p* < .001, η^2^ = .12). Post‐hoc comparisons for the main effect of Frequency showed that the mean simultaneity rate for AV stimuli pulsing at 1 Hz (*M* = 0.61, SE = 0.02) was significantly higher than for stimuli pulsing at 2 Hz (*M* = 0.47, SE = 0.01; *t*_(87)_ = 11.38, *p*_corr_ < .001) and at 3 Hz (*M* = 0.42, SE = 0.01; *t*_(87)_ = 11.39, *p*_corr_ < .001); the mean simultaneity rate also differed between stimuli presented at 2 and 3 Hz (*t*_(87)_ = 4.92, *p*_corr_ < .001). The main effect of SOA revealed that the proportion of simultaneous responses varied as a function of temporal asynchrony between stimuli, with the highest simultaneity rates for pulsing AV stimuli with temporal intervals close to +100 ms (see Figure 2a). The Frequency × SOA interaction will be explored in more detail in the following analyses on Gaussian‐ and Logistic‐derived parameters.

Coherently with the results of the rm‐ANOVA on simultaneity rates, the second rm‐ANOVA on TBWs revealed a main effect of Frequency (*F*_(1.21,105.13)_ = 77.17, *p* < .001, η^2^ = .47). Post‐hoc comparisons showed that participants' TBWs shrank with AV stimuli pulsing at 3 Hz (*M* = 273.11 ms, SE = 8.75 ms) as compared to 2 Hz (*M* = 307.19 ms, SE = 7.06 ms; *t*_(87)_ = −4.72, *p*_corr_ < .001) and 1 Hz (*M* = 458.41 ms, SE = 20.84 ms; *t*_(87)_ = −9.41, *p*_corr_ < .001); it also shrank for stimuli pulsing at 2 Hz as compared to those pulsing at 1 Hz (*t*_(87)_ = −8.49, *p*_corr_ < .001; see Figure 2a).

Regarding the analyses where SJ rates were analysed as a function of the leading sense, the rm‐ANOVA on slopes revealed a main effect of Frequency (*F*_(1.81,130.31)_ = 14.36, *p* < .001, η^2^ = .17) while the effect of Leading sense (*p* = .6) and the interaction (*p* = .3) between the two factors were not significant. Post hoc comparisons showed that participants had higher slope values (thus steeper logistic curves) when the AV stimuli were presented at 3 Hz (*M* = 0.08, SE = 0.01) as compared to stimuli presented at 1 Hz (*M* = 0.04, SE = 0.00; *t*_(72)_ = 5.83; *p*_corr_ < .001); slopes were also found to be higher for stimuli presented at 2 Hz (*M* = 0.06, SE = 0.01) as compared to those presented at 1 Hz (*t*_(72)_ = 3.47, *p*_corr_ < .005); slope values did not differ between stimuli presented at 3 Hz as compared to those presented at 2 Hz (*p*_corr_ = .2; see Figure 2b).

The last rm‐ANOVA on thresholds showed a main effect of Frequency (*F*_(1.67,120.02)_ = 68.47, *p*_corr_ < .001, η^2^ = .49) and a main effect of Leading sense (*F*_(1,72)_ = 398.24, *p* < .001, η^2^ = .85) while the Frequency × Leading sense interaction did not reach significance (*p* = .2). Post‐hoc comparisons for the main effect of frequency showed that participants had lower thresholds with AV stimuli pulsing at 3 Hz (*M* = 224.61 ms, SE = 7.66 ms) as compared to stimuli pulsing at 2 Hz (*M* = 253.04 ms, SE = 6.85 ms; *t*_(72)_ = −3.90, *p*_corr_ < .001) and at 1 Hz (*M* = 327.31 ms, SE = 9.27 ms; *t*_(72)_ = −9.53, *p*_corr_ < .001); thresholds were also found to significantly differ between stimuli pulsing at 2 Hz as compared to those pulsing at 1 Hz (*t*_(72)_ = −8.55, *p*_corr_ < .001). Post hoc comparisons for the main effect of Leading sense revealed that when auditory stimuli were presented first (i.e., AL conditions) participants' thresholds were significantly lower (*M* = 165.22 ms, SE = 7.79 ms) compared with VL conditions (*M* = 371.42 ms, SE = 8.10 ms; *t*_(72)_ = −19.96, *p*_corr_ < .001), in which visual stimuli preceded the auditory ones (see Figure 2b).

In Experiment 1, results show a reduction of the TBW as the frequency of the AV streams increases. Moreover, temporal acuity is higher when the onset of auditory streams temporally precedes the onset of visual streams, as compared to when visual streams are presented first.

## EXPERIMENT 2

In Experiment 2, we tested whether the shrinkage of TBWs found in Experiment 1 at higher stimulus frequencies could be due to the higher amount of information conveyed by the different AV streams. In fact, as previously mentioned, the number of pulses of AV stimuli in Experiment 1 was 3, 6 and 9 for the 1, 2 and 3 Hz conditions, respectively. The different number of pulses might have determined a more accurate judgment of simultaneity. To exclude this potential confound, here we kept the number of pulses constant (i.e., 3 pulses) across the three frequencies and manipulated instead the duration of the AV streams.

### Materials and methods

#### Participants

A new sample of 52 participants (28 F, mean age = 23.75, SD = 3.05, age range = 20–37) was recruited for Experiment 2, considering the same inclusion/exclusion criteria of Experiment 1. None of them took part in Experiment 1.

#### Stimuli and procedure

We employed the same AV stimuli and the same procedures of Experiment 1. The only difference was that we equalized the number of pulses (*n* = 3) for the three different frequencies of AV streams. By doing so, we obtained different durations of the audio‐visual streams: stimuli presented at 1 Hz had a duration of 3 s, which decreased to 1.5 s and to 1 s in the 2 and 3 Hz conditions, respectively.

### Data analysis

In Experiment 2 we employed the same data analyses used in Experiment 1.

### Results

Among the 52 participants who completed the task, data from two subjects were excluded from the analyses on TBWs and SJ rates because of a poor fitting of the Gaussian function (adjusted *R* ^2^ < .5). Thus, the final sample included 50 participants (28 F, mean age = 23.76, SD = 3.07, age range = 20–37). For the analyses on slopes and thresholds, data from 16 participants were instead excluded because of a poor logistic fitting (i.e., adjusted *R* ^2^ < .3 in either the AL or VL condition). Thus, the final sample included 36 participants (20 F, Mean Age = 24.44, SD = 3.31, Age Range = 21–37).

**FIGURE 3:**
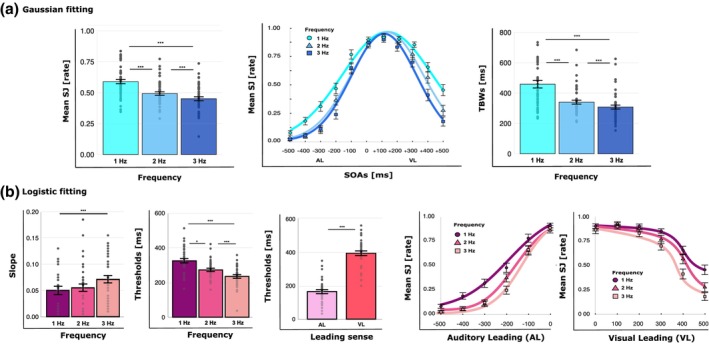
Experiment 2 results. (a) Gaussian fitting results: Mean simultaneity judgements (SJ) and the temporal binding windows (TBWs) with error bars (SEM) and individual data points for each stimulation frequency (1 Hz = cyan, 2 Hz = light blue, 3 Hz = dark blue) and SOA (1 Hz = circles, 2 Hz = triangles, 3 Hz = squares). (b) Logistic fitting results: Mean slope and threshold values with error bars (SEM) and individual data points for each stimulation frequency (1 Hz = violet, 2 Hz = fuchsia, 3 Hz = dark pink), leading sense (AL = light pink, VL = red), and SOA (1 Hz = circles, 2 Hz = triangles, 3 Hz = squares). Asterisks indicate the level of statistical significance (***p < .001, **p < .01).

The results replicated findings of Experiment 1. Indeed, the rm‐ANOVA on SJ rates showed a main effect of frequency (*F*_(1.57,77.72)_ = 41.92, *p* < .001, η^2^ = .46), SOA (*F*_(3.19,156.41)_ = 177.96, *p* < .001, η^2^ = .78) and a significant frequency × SOA interaction (*F*_(12.13,594.39)_ = 4.20, *p* < .001, η^2^ = .08). Post hoc comparisons for the main effect of frequency showed that the mean simultaneity rate for AV stimuli pulsing at 1 Hz (*M* = 0.59, SE = 0.02) was significantly higher than for stimuli pulsing at 2 Hz (*M* = 0.49, SE = 0.01; *t*_(49)_ = 6.04, *p*_corr_ < .001) and at 3 Hz (*M* = 0.45, SE = 0.02; *t*_(49)_ = 7.52, *p*_corr_ < .001); the mean simultaneity rate also differed between stimuli presented at 2 Hz and at 3 Hz (*t*_(49)_ = 3.73, *p*_corr_ < .005). As for Experiment 1, the main effect of SOA revealed that participants reported significantly higher SJ rates when dynamic AV stimuli were presented at temporal intervals closer to +100 ms (VL; see Figure 3a). The frequency × SOA interaction will be explored in more detail with subsequent analyses.

The second rm‐ANOVA on TBWs revealed a main effect of frequency (*F*_(1.24,60.57)_ = 36.65, *p* < .001, η^2^ = .42). Post hoc *t*‐tests showed that participants' TBWs shrank with stimuli pulsing at 3 Hz (*M* = 307.79 ms, SE = 13.09 ms) as compared to 2 Hz (*M* = 340.06 ms, SE = 12.66 ms; *t*_(49)_ = −3.62, *p*_corr_ < .005) and 1 Hz (*M* = 458.50 ms, SE = 25.03 ms; *t*_(49)_ = −6.55, *p*_corr_ < .001); it also shrank for stimuli pulsing at 2 Hz as compared to those pulsing at 1 Hz (*t*_(49)_ = −5.58, *p*_corr_ < .001; see Figure 3a).

Regarding the analyses where SJ rates were analysed as a function of the leading sense, the rm‐ANOVA on slopes revealed only a main effect of frequency (*F*_(1.99,69.84)_ = 3.71, *p* < .05, η^2^ = .10), while Leading sense and the frequency × leading sense interaction did not reach significance (*p* = .2 and .6, respectively). Post hoc comparisons for the main effect of frequency showed that participants had higher slope values (steeper logistic curves) when the AV stimuli were presented at 3 Hz (*M* = 0.07, SE = 0.01) as compared to stimuli presented at 1 Hz (0.05, SE = 0.01; *t*_(35)_ = 2.56, *p*_corr_ < .05); in this case, slope values did not differ between stimuli presented at 2 Hz as compared to those presented at 1 Hz (*p*_corr_ = 1.0) and 3 Hz (*p*_corr_ = .1; see Figure 3b).

The last rm‐ANOVA on thresholds showed a main effect of Frequency (*F*_(1.54,53.92)_ = 32.03, *p* < .001, η^2^ = .48), a main effect of Leading sense (*F*_(1,35)_ = 141.42, *p* < .001, *η* ^2^ = .80) and a significant frequency × leading sense interaction (*F*_(2,70)_ = 3.29, *p* < .05, η^2^ = .10). Post‐hoc comparisons for the main effect of frequency showed that participants had lower thresholds with AV stimuli pulsing at 3 Hz (*M* = 234.41 ms, SE = 10.23 ms) as compared to stimuli pulsing at 2 Hz (*M* = 272.02 ms, SE = 9.36 ms; *t*_(35)_ = −4.49, *p*_corr_ < .001) and 1 Hz (*M* = 324.46 ms, SE = 12.30 ms; *t*_(35)_ = −6.52, *p*_corr_ < .001); thresholds were also found to significantly differ between stimuli pulsing at 2 Hz as compared to those pulsing at 1 Hz (*t*_(35)_ = −4.74, *p*_corr_ < .001). Post hoc comparisons for the main effect of Leading sense revealed that when auditory stimuli were presented first (AL conditions) participants' thresholds were significantly lower (*M* = 163.15 ms, SE = 11.64 ms) compared with conditions in which visual stimuli preceded the auditory ones (VL conditions; *M* = 384.40 ms, SE = 13.82 ms; *t*_(35)_ = −11.89, *p*_corr_ < .001). In order to further explore the interaction between these two factors, we performed post hoc tests comparing thresholds for the three stimulation frequencies in the AL and VL conditions. In particular, when visual streams were presented before the auditory ones (VL condition), participants reported lower thresholds for stimuli pulsing at 3 Hz (*M* = 334.71 ms, SE = 17.65 ms) as compared to stimuli presented at 2 Hz (*M* = 397.50 ms, SE = 16.47 ms, *t*_(35)_ = −4.35, *p*_corr_ < .001) and 1 Hz (*M* = 440.10 ms, SE = 15.00 ms, *t*_(35)_ = −6.28, *p*_corr_ < .001); thresholds were also found to significantly differ between stimuli pulsing at 2 Hz as compared to those pulsing at 1 Hz (*t*_(35)_ = −2.94, *p*_corr_ < .05). When looking at the AL condition, participants reported lower thresholds for stimuli pulsing at 3 Hz (*M* = 134.12 ms, SE = 13.86 ms) as compared to stimuli presented at 1 Hz (*M* = 208.81 ms, SE = 16.4 ms, *t*_(35)_ = −4.29, *p*_corr_ < .001) and between stimuli pulsing at 1 and 2 Hz (*M* = 146.53 ms, SE = 12.67, *t*_(35)_ = 4.27, *p*_corr_ < .001), while no differences were found among thresholds for stimuli pulsing at 2 and 3 Hz (*p*_corr_ = 1.00; see Figure 3b).

The pattern emerging in Experiment 2 confirms the results of Experiment 1 and also suggests that the quantity of information, equalized for the number of cycles across the different frequencies, did not impact temporal integration.

## EXPERIMENT 3

In previous experiments, we found that the frequency of AV stimuli presentation impacts multisensory integration, determining higher temporal acuity (i.e., less AV integration) with increasing stimulation frequency, regardless of the number of pulses (Experiment 1) and/or the duration of the AV streams (Experiment 2). Stimuli employed in both previous experiments were created following a pure sinusoidal function, and thus they were carrying a purely regular rhythm.

The rationale of Experiment 3 was to investigate whether the quasi‐rhythmic properties of speech could influence the extension of TBWs. Specifically, we hypothesized that speech‐like AV stimuli might be perceived as synchronous for extended time intervals when compared to pure regular stimuli. Such extended TBWs would suggest that the quasi‐rhythmic features of a typical speech envelope could influence AV temporal acuity. To this aim, we compared SJ rates and TBWs extensions for irregular (quasi‐rhythmic) AV stimuli modulated in amplitude/size following a complex function obtained from the speech envelopes of real speech signals. As comparison stimuli, we used 3 Hz rhythmic stimuli, the same as those used in the previous two experiments, given that this was comparable in terms of the main frequency present in the signal.

### Materials and methods

#### Participants

A new sample of 36 participants (21 F, mean age = 21.83, SD = 2.07, age range = 18–27) was recruited for Experiment 3, considering the same inclusion/exclusion criteria of Experiments 1 and 2. None of them took part in Experiment 1 or 2.

#### Stimuli and procedure

In the Rhythmic condition, continuous AV streams modulated following a regular sinusoidal function at 3 Hz and with a duration of 3 s (the same as in Experiment 1) were used. In the Speech‐like condition, AV stimuli were modulated following the quasi‐rhythmic pattern of a speech envelope. Speech envelopes were obtained from different spoken sentences taken from articles of the Italian Constitution, which were recorded by a female voice and lasted 3 s; subsequently, we applied a Fast Fourier Transform (FFT) of such speech envelopes in order to obtain the frequency power spectrum. We selected five sentences for which the final speech envelopes showed a clear peak at ~3 Hz and, subsequently, used the relative envelope to create our quasi‐rhythmic speech‐like AV stimuli (see Figure 1d,e).

The task procedure was the same as for Experiments 1 and 2. The total number of trials was 165 (5 repetitions for each combination of SOAs and frequency).

### Data analysis

In Experiment 3, we employed the same data analysis methods used in Experiments 1 and 2. A repeated rm‐ANOVA was conducted on SJ rates with condition (rhythmic and speech‐like) and SOA (−500, −400, −300, −200, −100, 0, +100, +200, +300, +400 and +500 ms) as within‐subject factors. A second rm‐ANOVA on TBWs was conducted using condition (rhythmic and speech‐like) as a within‐subject factor. Finally, two different rm‐ANOVAs on slopes and thresholds extracted from the logistic fittings were performed with condition (rhythmic and speech‐like) and leading sense (AL and VL) as within‐subject factors.

### Results

Among the 36 participants who completed the task, data from two subjects were excluded from the analyses on SJ rates and TBWs because of a poor fitting of the Gaussian function (adjusted *R* ^2^ < .5). The final sample included 34 participants (20 F, mean age = 21.85, SD = 2.13, age range = 18–27). For the analyses on slopes and thresholds, data from eight participants were instead excluded because of a poor logistic fitting (adjusted *R* ^2^ < .3 in either the AL or VL condition). The final sample included 28 participants (16 F, mean age = 22.00, SD = 2.93, age range = 18–27).

**FIGURE 4:**
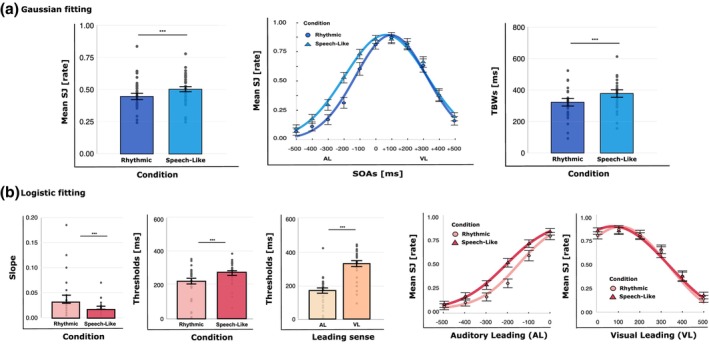
Experiment 3 results. (a) Gaussian fitting results: Mean simultaneity judgements (SJ) and the temporal binding windows (TBWs) with error bars (SEM) and individual data points for each condition (rhythmic = dark blue, speech‐like = medium blue) and SOA (rhythmic = circles, speech‐like = triangles). (b) Logistic fitting results: Mean slope and threshold values with error bars (SEM) and individual data points for condition (rhythmic = light pink, speech‐like = red), leading sense (AL = light yellow, VL = orange) and SOA (rhythmic = circles, speech‐like = triangles). Asterisks indicate the level of statistical significance (***p < .001, **p < .01).

The rm‐ANOVA on SJ rates showed a main effect of condition (*F*_(1,33)_ = 10.61, *p* < .005, η^2^ = .24), SOA (*F*_(2.95,97.35)_ = 76.82, *p* < .001, η^2^ = .70) and a significant condition × SOA interaction (*F*_(6.64,219.11)_ = 4.83, *p* < .001, η^2^ = .13). Post hoc comparisons for the main effect of condition showed that the mean SJ rate for AV streams pulsing with a speech‐like modulation pattern (*M* = 0.50, SE = 0.02) was significantly higher than for streams pulsing with a rhythmic pattern (*M* = 0.45, SE = 0.02; *t*_(33)_ = 3.26, *p*_corr_ <.005). As for Experiments 1 and 2, the main effect of SOA revealed that the proportion of simultaneous responses varied as a function of temporal asynchrony between streams (see Figure 4a). The condition × SOA interaction will be explored in more detail with subsequent analyses.

The second rm‐ANOVA on TBWs revealed a main effect of condition (*F*_(1,33)_ = 39.20, *p* < .001, η^2^ = .54). Post hoc *t*‐tests showed that participants' TBWs were enlarged when processing speech‐like streams (*M* = 373.10 ms, SE = 23.46 ms) as compared to streams with a regular rhythm (*M* = 318.23, SE = 24.10 ms; *t*_(33)_ = 6.26, *p* < .001; see Figure 4a).

Regarding the analyses where SJ were analysed as a function of the leading sense, the rm‐ANOVA on slopes revealed a main effect of condition (*F*_(1,27)_ = 5.45, *p* < .05, η^2^ = .17). Post‐hoc comparisons showed that participants had higher slope values, representing steeper logistic curves for rhythmic AV streams (*M* = 0.04, SE = 0.01) as compared with speech‐like AV streams (*M* = 0.02, SE = 0.00; *p* < .001; see Figure 4b). Leading sense and the condition × leading sense interaction did not reach significance (*p* > .2).

The last rm‐ANOVA on thresholds showed a main effect of condition (*F*_(1,27)_ = 13.07, *p* < .005, η^2^ = .32) a main effect of leading sense (*F*_(1,27)_ = 75.10, *p* < .001, η^2^ = .74) and a significant condition × leading sense interaction (*F*_(1,27)_ = 11.81, *p* < .005, η^2^ = .30). Post hoc comparisons for the main effect of condition showed that participants had lower thresholds for rhythmic AV streams (*M* = 225.77 ms, SE = 17.06 ms) as compared to Speech‐like AV streams (*M* = 274.19 ms, SE = 13.70 ms; *t*_(27)_ = −3.61, *p*_corr_ < .005). Post‐hoc comparisons for the main effect of Leading sense revealed that in the AL condition, thresholds were significantly lower (*M* = 169.67 ms, SE = 15.80 ms) compared with VL conditions (*M* = 330.29 ms, SE = 17.75 ms; *t*_(27)_ = −8.67, *p*_corr_ < .005). Importantly, concerning the interaction between condition and leading sense, post‐hoc comparisons showed that thresholds significantly differ between rhythmic (*M* = 128.71 ms, SE = 16.38 ms) and speech‐like AV streams (*M* = 210.63 ms, SE = 17.29 ms) only in the AL condition (*t*_(27)_ = −7.01, *p*_corr_ < .005), while no differences were found in the VL condition (*p*_corr_ = .94; see Figure 4b).

Results of Experiment 3 show more tolerant temporal integration for the speech‐like condition with respect to a purely rhythmic condition with a comparable ‘average’ frequency.

## DISCUSSION

Increasing evidence shows that the spatiotemporal relationship between multisensory stimuli is fundamental in determining whether information from cross‐modal sources would be integrated into a unified percept (Recanzone 2009; Fain, 2003; Spence, 2007; Stein & Stanford, 2008). These mechanisms have been widely investigated within the audio‐visual domain by employing simple discrete stimuli, like flash‐beep or complex speech signals (Conrey & Pisoni, 2006; Miller & D'esposito, 2005; Stevenson & Wallace, 2013; van Atteveldt et al., 2007; Van Wassenhove et al., 2007; Vatakis & Spence, 2008). The main findings, largely explored through the temporal binding windows account, suggest that simple basic stimuli are integrated into narrower temporal windows, compared to more complex AV pairs, which are characterized by a richer temporal structure (Denison et al., 2013). Although these results appear to be consistent across multiple studies, the key features that account for this complexity are less clear. Since speech stimuli, like complex natural inputs in general, are characterized by continuous and rhythmic or quasi‐rhythmic structures, these characteristics might be suitable candidates for explaining the complexity that determines higher temporal tolerance to asynchronies across modalities and, in turn, the widening of TBW. By systematically manipulating the frequency and rhythmic structure of AV streams across three experiments, we aimed at investigating whether and how the spectro‐temporal profile of AV stimuli drives temporal integration/segregation.

Specifically, auditory and visual stimuli were modulated at different temporal frequencies (1, 2 and 3 Hz) and presented simultaneously (SOA = 0 ms) or with varying stimulus onset asynchronies (from −500 to +500 ms, with negative SOAs indicating that the auditory stimulus preceded the visual one, while positive SOAs indicate the opposite). In Experiment 1, AV streams were matched in duration (3 s) and only differed in the number of pulses (i.e., 3, 6 or 9 for the 1, 2 and 3 Hz conditions, respectively). In Experiment 2, a complementary approach was adopted: the total number of pulses was kept constant (i.e., 3 pulses) across the three frequencies while the duration of the AV streams varied (i.e., 1 Hz = 3 s, 2 Hz = 1.5 s and 3 Hz = 1 s). Lastly, in Experiment 3, AV stimuli were modulated following purely rhythmic vs. quasi‐rhythmic (speech‐like) envelopes while the dominant stimulation frequency was kept constant (3 Hz). Gaussian and logistic fitting‐derived parameters allowed for the estimation of ‘summary’ measures of audio‐visual temporal processing, less sensitive to random noise and trial‐by‐trial variability compared to the raw SJ data points. In this framework, differences between auditory‐ and visual‐leading conditions, and how they vary with frequency, enabled us to more precisely capture shifts in integration boundaries and asymmetries in sensory processing.

First, we tested whether the rhythmic pattern of a congruent AV stream could impact the individual perception of synchrony and AV temporal acuity, measured as the difference in TBWs size as a function of the AV streams frequency. We hypothesized that streams pulsing at higher frequencies would generally determine shorter integration windows (higher AV temporal acuity). In line with our expectations, the results of Experiments 1 and 2 suggest that TBWs shrink as the stimulus frequency increases, even when the AV streams are equalized for the number of pulses across the different frequencies. This trend is evident not only in the TBW estimates but also in threshold and slope values. Indeed, all measures derived from Gaussian and logistic fits show a consistent and converging pattern: as the modulation frequency of the AV stream increases, so does the temporal precision of cross‐modal integration. This is reflected in narrower TBWs, steeper psychometric slopes and lower thresholds, all indicative of greater temporal acuity. Such consistency across distinct outcomes strengthens the robustness of our findings and provides converging evidence that frequency‐dependent changes in AV integration are not limited to one specific measure but generalize across different facets of temporal processing.

A robust and well‐documented phenomenon in multisensory temporal perception is a bias toward perceiving visual‐leading stimuli as more synchronous than auditory‐leading ones, reflecting the brain's greater tolerance for visual delays relative to auditory ones when judging simultaneity (Fain, 2003; Spence, 2007; Vroomen & Keetels, 2010). The skewness may arise from a combination of factors such as ecological tuning to audio‐visual events in the environment, attentionally mediated differences in temporal resolution or salience and individual differences in temporal sensitivity or sensory dominance (Marsicano et al., 2022; Stevenson et al., 2012). Our findings from both Experiment 1 and Experiment 2 suggest that frequency compression or expansion of AV streams does not alter the relative dominance or perceptual advantage of one leading modality over the other. Instead, frequency acts as a global modulating factor that shifts thresholds overall, while the asymmetry favouring the visual‐leading modality remains consistent across conditions. Thus, frequency influences general temporal precision (reflected in narrower TBWs and lower thresholds) but does not differentially affect AL and VL conditions.

A previous study conducted by Arrighi et al. (2006) found that the perception of AV synchrony of a naturalistic scene with a drumming player was modulated by the drumming tempos. Specifically, when comparing the TBWs for slow series, with drum rhythm variations between 1 and 4 Hz and fast series with drum variations between 4 and 11 Hz, they found that higher drumming tempos are integrated into narrower TBWs compared to the lower frequency rhythms, suggesting that faster frequencies allow for an increased temporal discrimination. This frequency‐dependent modulation of temporal acuity is also compatible with the electrophysiological properties of motion‐sensitive visual areas (Bair & Movshon, 2004; Battaglini et al., 2020; Di Dona & Ronconi, 2023; Ronconi, Balestrieri, et al., 2023). For instance, neural responses to moving stimuli vary systematically with temporal frequency in the middle temporal (MT) area of the extrastriate visual cortex. At low temporal frequencies, responses were broadly distributed in time, with a long latency to peak response (approximately 80 ms). At high temporal frequencies, the response is narrower in time with a shorter latency to peak response (30–40 ms) (Arrighi et al., 2006). These previous observations suggest that with increased stimulation frequency the temporal precision of sensory encoding is enhanced, which in turn may facilitate more fine‐grained temporal judgements, consistent with the behavioural narrowing of TBWs observed in our data.

After having established in Experiments 1 and 2 that the frequency (speed) of AV streams clearly influences the size of cross‐modal integration windows, in Experiment 3 we moved a step forward and tested whether the spectral properties of speech, characterized by irregular (or quasi‐regular) rhythms, would account for larger TBWs even when the linguistic nature of the auditory input was taken apart. Thus, with this additional experiment we could precisely test the hypothesis that the inherent irregularity, or the decrease in regularity, would be one of the primary factors that determine the widening of TBWs size obtained when using AV speech as compared to other non‐linguistic complex AV streams. Three‐second speech stimuli with a clear peak at 3 Hz were selected from different recordings of a female voice reading short sentences, and their relative envelope was used to modulate the AV streams.

We expected that when comparing regular, sinusoidally modulated AV stimuli with quasi‐rhythmic AV stimuli modulated following a speech envelope, such a spectral profile would account for the widening of TBW, even in the absence of a linguistic message. Results from Experiment 3 confirmed the hypothesis, showing that the irregular/quasi‐rhythmic nature of speech‐like stimuli was integrated across a wider TBW; however, this was evident only in AL trials, when the auditory stream preceded the visual stream. This selective effect is particularly notable, as it contrasts with the typical visual‐leading advantage, indicating that speech‐like temporal structures may mitigate, or even eliminate, the usual perceptual bias toward visual‐leading asynchronies. This result highlights an interplay between stimulus‐driven temporal dynamics and perceptual tuning shaped by ecological and neural constraints, emphasizing how spectral variability, common in naturalistic AV input such as speech, can modulate the limits of cross‐modal temporal integration.

One factor that could, in principle, influence simultaneity judgements (and thus TBWs estimation) is the instantaneous phase difference between auditory and visual signals, particularly when considering amplitude alignment at specific stimulus onsets. However, our temporally extended AV streams (lasting between 1.5 and 3 s) contained multiple pulses (3, 6 or 9 depending on frequency), rather than single onset events. Thus, judgements of synchrony were likely based on the perceived coherence of the full temporal structure of the audio‐visual streams rather than on momentary amplitude differences or on the alignment of the first AV pulses. Indeed, if perceptual integration were driven primarily by initial phase differences or pulse‐onset alignment, it would not have been possible to see differences among the different stream frequencies in Experiments 1 and 2. Moreover, data from Experiment 3 show that the increased temporal tolerance observed for speech‐like stimuli was primarily auditory‐driven. If instantaneous phase differences were responsible for the observed effects, we would expect them to affect simultaneity judgements equally in both auditory‐ and visual‐leading conditions.

While previous studies have highlighted the variable influence of speech on temporal processing (Bishop & Miller, 2009; Grant & Seitz, 2000; Munhall & Vatikiotis‐Bateson, 2004; Van Wassenhove et al., 2007; Vatakis et al., 2008; Vatakis & Spence, 2008; Venezia et al., 2016; Vroomen & Stekelenburg, 2011), the novelty of our work lies in the direct comparison between strictly rhythmic and quasi‐rhythmic (speech‐like) streams, with matched dominant frequencies (3 Hz). By holding stimulus frequency constant, we specifically isolate the effect of envelope regularity on AV integration/integration and showed that speech‐like envelopes (even in the absence of any linguistic content) induce broader and asymmetric TBWs, favouring auditory‐leading asynchronies. These results indicate that the spectrotemporal variability of naturalistic stimuli modulates temporal binding beyond frequency content alone. Indeed, the brain's tolerance for temporal discrepancies is not only frequency‐dependent but also sensitive to the dynamic variability of sensory inputs. It is important to note that in Exp. 3 we opted to match the rhythmic and quasi‐rhythmic streams based on the mean dominant frequency (3 Hz), without explicitly controlling for other potentially relevant dimensions such as phase structure and spectrotemporal complexity. While this approach was theoretically motivated as a first step investigation, future work should aim to refine this comparison by more thoroughly matching the temporal and spectral characteristics of experimental and control stimuli to better isolate the influence of higher‐order features intrinsic to speech‐like inputs.

Overall, we believe that the findings we observed across the three experiments reinforce the idea that the cross‐modal influences in sensory cortices are mediated by the synchronization of ongoing neural oscillations, in particular phase‐reset and neural entrainment, which would play a central role not only in influencing the human ability to track and perceive AV streams with different spectral properties, but also to track multimodal speech signals (Bauer et al., 2020; Peelle & Davis, 2012; Zoefel et al., 2018). In this regard, the phase of oscillatory activity can strongly impact the effectiveness of perception and behaviour as it reflects the excitability states of the involved neuronal ensembles (Bauer et al., 2020). The correspondence of the onset of sensory inputs with high‐excitability states promotes stronger neural responses while the opposite applies to low‐excitability states. Consequently, as shown by several psychophysics studies, behavioural performance is enhanced when the stimulus onset falls in a preferential high‐excitability state across sensory modalities (Contemori et al., 2022; Fiebelkorn et al., 2013; Helfrich et al., 2018; Landau & Fries, 2012; Ronconi & Bellacosa Marotti, 2017; Ronconi & Melcher, 2017).

In the context of multisensory processing, in particular, it has been widely demonstrated that phase reset is a key mechanism for AV integration, given that both auditory and visual stimuli can cross‐modally ‘realign’ the phase of oscillatory activity in the visual and auditory cortex, respectively (Lakatos et al., 2009; Mercier et al., 2013; Romei et al., 2012; Ronconi, Vitale, et al., 2023). This cross‐modal realignment would be important for aligning rhythmic brain activity of auditory and visual areas and for opening a temporal window where audio‐visual integration can occur (Bauer et al., 2020). The neural pathways for generating cross‐modal phase reset are only partially understood, but preliminary evidence supports multiple hypotheses: there can be direct lateral connections between unimodal cortices (Falchier et al., 2002), cross‐modal influences through higher‐order multimodal cortical regions (superior temporal sulcus, intraparietal sulcus and prefrontal cortex) (Driver & Noesselt, 2008; Ghazanfar & Schroeder, 2006; van Atteveldt et al., 2014), or even pathways involving multimodal subcortical regions (e.g., superior colliculus and thalamus) (Cappe et al., 2007; Hackett et al., 2007; Lakatos et al., 2007). Their differential involvement may depend on the specific stimulus parameters or task demands and also on the exploitation of top‐down factors.

The main findings of Experiments 1 and 2, that is, that higher frequencies in the AV streams led to narrower TBWs and so higher temporal acuity, could be explained by phase reset mechanisms of ongoing neural oscillations. The presentation of transient stimuli, indeed, determines a phase reset of ongoing neural oscillations in primary sensory cortices, leading to the temporal reorganization of neural high‐excitability states to optimally respond to the environment (Bauer et al., 2020). The series of phase resets that rhythmic AV streams induce in neural oscillations ultimately leads to neuronal entrainment of multisensory‐sensitive regions, and might be at the basis of our behavioural findings. Specifically, stimuli varying at higher frequencies could putatively induce a higher number of phase resets per time unit, determining a higher number of realignments of ongoing oscillations to high‐excitability states, ultimately leading to more precise temporal processing and thus narrower TBWs.

Considering the effects emerging in Experiments 1 and 2 and their presumed associations with cross‐modal phase resets and/or neural entrainment, two AV streams pulsing at the same ‘average’ frequency should lead to similar TBW or temporal acuity. Instead, in Exp. 3, an asymmetrical widening of the TBW was observed for the speech‐like AV stream when compared with a purely rhythmic AV stream, despite the average dominant frequency being the same (i.e., 3 Hz). In particular, while for the VL condition, the psychometric fits are very similar and almost superimposed, for the AL conditions, a lower temporal acuity (i.e., a more tolerant integration window) was observed for speech‐like modulated streams, as suggested by the higher threshold of the logistic fit. One possibility is that, despite the average dominant frequency being comparable across conditions, the spectral complexity embedded in the speech‐like streams, in which both lower and higher frequency components were still present, may have induced different temporal acuity across conditions. However, if this consideration holds, it would be reasonable to expect a threshold modulation not only in the AL but also in the VL condition.

Another possibility revolves around the information mostly conveyed by the auditory stream. In this regard, the auditory stream was created by modulating the amplitude of a pure tone following the envelope of a natural speech recording. While such a stimulus holds no purely linguistic information, it retains a very similar spectrotemporal structure. Previous studies have shown that stimuli resembling speech from which linguistic features were removed (e.g., spectrally rotated speech (Di Dona et al., 2022; Marklund et al., 2020; Steinmetzger & Rosen, 2017) or temporally reversed speech (Ishida, 2021; Mai & Wang, 2023)) led to larger temporal integration windows (Maier et al., 2011; Shahin et al., 2017). Stimuli with spectrotemporal patterns similar to speech may trigger perceptual modes typical of speech processing and perception, by which larger integration windows allow for the integration of faster (e.g., phonemes and syllables) and slower (e.g., sentences and prosodic structures) unfolding information to properly understand the meaning of utterances (Ding et al., 2016; Keitel et al., 2018; Martin, 2020). While visual information is also very important for effective speech perception (Mcgurk & Macdonald, 1976), as well as its integration with auditory information (Marques et al., 2016; Peelle & Sommers, 2015), the auditory modality is undoubtedly self‐sufficient for speech perception. Importantly, the visual part of the AV streams employed in the present study was created by adapting the same envelope used for the auditory one, but it was used to modulate the radius of a pulsing circle in order to resemble a moving mouth. However, it is likely that this impoverished implementation did not sufficiently mimic the movements of a speaking mouth to activate the perceptual modalities typical of speech perception. In fact, the movement of a speaking mouth is not completely coherent with the auditory envelope; its profile also depends on many different factors such as movements of unseen body parts (e.g., tongue, vocal cords and larynx) or other physical features of the mouth which we did not consider. In this regard, previous studies employing natural AV stimuli (Marques et al., 2016; Mcgurk & Macdonald, 1976; Peelle & Sommers, 2015) could presumably activate motion‐sensitive and/or face‐specific cortical sites which might have contributed to the activation of the perceptual models typical of speech perception. Therefore, in the VL condition, this modulation might not have triggered processing modes typical of speech perception as the AL condition possibly did, resulting in no difference in terms of temporal integration between the speech‐like and the rhythmic conditions.

A third possibility relates to innate sensory biases or evolved asymmetries in cross‐modal processing of speech‐like streams. Speech communication is characterized by temporal disparities, where articulatory gestures may lag slightly behind their corresponding acoustic cues and vice versa, due to biomechanical and neural delays (Fain, 2003; Murray et al., 2016; Pasqualotto et al., 2016). As a result, the human brain might have evolved a greater tolerance, or a broader temporal binding window, for auditory‐leading asynchronies when the signal carries speech‐like information. This asymmetry could reflect an ecological adaptation, optimizing integration in realistic communicative settings. The lack of an analogous widening in the VL condition supports the idea that this flexibility reflects modality‐specific temporal expectations, putatively shaped by the statistics of natural sensory environments.

To summarize, the present work showed that the integration of dynamic AV stream is modulated by their rhythmicity: streams with higher frequency might trigger a higher number of phase resets per time unit, determining a higher number of realignments of ongoing oscillations to high‐excitability states, ultimately leading to more precise temporal processing and thus narrower TBWs. Furthermore, we showed that pulsing AV stimuli with an auditory spectrotemporal profile resembling speech may trigger perceptual modes typical of speech perception, leading to reduced temporal acuity and increased temporal integration when the auditory information is presented slightly before the visual one. Thus, both low‐and high‐level spectrotemporal information influence AV temporal integration of continuous streams.

In most real‐world contexts, the brain does not operate at a single dominant oscillatory frequency, but rather exhibits simultaneous activity across multiple frequency bands, each linked to distinct functional processes (Meyer et al., 2020; Ronconi, Balestrieri, et al., 2023; Zoefel et al., 2018). For instance, phase‐amplitude coupling between slower (e.g., delta/theta) and faster (e.g., beta/gamma) rhythms has been proposed as a mechanism that facilitates dynamic coordination and information exchange between sensory and higher‐order cortical regions (Ronconi, Balestrieri, et al., 2023). This mechanism may be especially relevant for integrating multisensory input that unfolds at different temporal scales, such as auditory and visual components of naturalistic streams. In this framework, lower‐frequency oscillations could support the tracking of slow global dynamics (e.g., prosody and syllabic structure), while higher‐frequency activity might encode finer temporal details (e.g., phonemes or rapid visual transients), thus enabling a temporally nested representation of cross‐modal events (Bauer et al., 2020; Meyer et al., 2020; Peelle & Davis, 2012; Poeppel & Assaneo, 2020). The observed modulation of TBW by both stream frequency and rhythmic regularity in our experiments might reflect how cross‐modal phase reset and entrainment operate within such a multiplexed scenario. Quasi‐rhythmic, speech‐like AV streams may disrupt optimal cross‐frequency alignment or impose a greater demand on hierarchical temporal integration, leading to wider integration windows.

Future work using electrophysiological techniques could help delineate how specific phase‐amplitude coupling profiles support the integration of complex, temporally structured AV signals and whether these dynamics are altered in clinical populations previously found to be characterized by dysfunctional integrative mechanisms such as autism, schizophrenia and dyslexia (Ferri et al., 2017; Hairston et al., 2005; Marsicano et al., 2022, 2025; Ronconi, Vitale, et al., 2023). Indeed, individuals with ASD have been shown to be characterized by enlarged TBWs, leading to a broader timeframe in which asynchronous sensory inputs are perceived as simultaneous (Ronconi, Vitale, et al., 2023). This diminished temporal acuity can impair the integration of audio‐visual speech cues, adversely affecting language comprehension and social interaction. Similarly, schizophrenia is associated with widened TBWs, resulting in the hyperintegration of temporally misaligned sensory information (Ferri et al., 2017). This aberrant integration may contribute to symptoms such as hallucinations and delusions by blurring the distinction between internal thoughts and external stimuli. In dyslexia, temporal processing deficits can hinder the precise alignment of auditory and visual inputs (Hairston et al., 2005), hereby affecting reading, comprehension and spelling abilities. These findings underscore the importance of TBW calibration for typical perceptual and cognitive development and suggest that its disruption may be a transdiagnostic marker of dysfunctional sensory integration. Understanding the role of altered TBWs in these disorders not only sheds light on their underlying neurocognitive mechanisms but also opens avenues for targeted interventions. Indeed, it could be investigated whether stimulus rhythmicity and envelope regularity could be manipulated to causally probe or even remediate atypical multisensory processing in these populations. Behavioural or neurostimulation‐based protocols using (quasi‐)rhythmic AV streams could potentially entrain neural oscillations to enhance multisensory acuity, paving the way for individualized interventions designed to restore optimal temporal integration/segregation dynamics.

## AUTHOR CONTRIBUTIONS

**Denisa Adina Zamfira:** Data curation; formal analysis; visualization; writing – original draft; writing – review and editing; software. **Giuseppe Di Dona:** Data curation; formal analysis; visualization; writing – original draft; writing – review and editing; software. **Gianluca Marsicano:** Methodology; investigation; software; validation; data curation; writing – review and editing. **Martina Battista:** Data curation; formal analysis; writing – original draft; writing – review and editing. **Luca Battaglini:** Conceptualization; methodology; supervision; writing – review and editing; resources. **Luca Ronconi:** Conceptualization; supervision; project administration; validation; funding acquisition; resources; methodology; writing – review and editing.

## FUNDING INFORMATION

D.A.Z., G.D.D., M.B. and L.R. were supported by funding from Fondazione Regionale per la Ricerca Biomedica of Regione Lombardia (FRRB Early Career Award ID: 1751150 to L.R.); G.D.D. and L.R. were supported by funding from the Italian Ministry of University and Research (Bando PRIN 2022 ID: 2022H4ZRSN to L.R.).

## CONFLICT OF INTEREST STATEMENT

The authors declare no competing interests.

## Data Availability

The aggregated data and analysis scripts that support the findings of this study are openly available at https://osf.io/bsgqd/?view_only=14f5e7382ebb485eb4ae25a0b65d9fa5.
